# Maternal infections during pregnancy and offspring midlife inflammation

**DOI:** 10.1186/s40748-019-0099-3

**Published:** 2019-03-14

**Authors:** Jolene Masters Pedersen, Erik Lykke Mortensen, Rikke Hodal Meincke, Gitte Lindved Petersen, Esben Budtz-Jørgensen, Helle Brunnsgaard, Holger Jelling Sørensen, Rikke Lund

**Affiliations:** 1 Section of Social Medicine, Department of Public Health, University of Copenhagen, Copenhagen, Denmark; 2 Center for Healthy Aging, University of Copenhagen, Copenhagen, Denmark; 3 Section of Environmental Health, Department of Public Health, University of Copenhagen, Copenhagen, Denmark; 4 Department of Clinical Immunology, Rigshospitalet, University Hospital of Copenhagen, Copenhagen, Denmark; 5 Mental Health Centre Copenhagen, Gentofte Hospital, Copenhagen, The Capital Region Denmark

**Keywords:** Infection, Inflammation

## Abstract

**Background:**

Microbial exposures early in life have been found to be associated with lower levels of inflammation in adulthood; however, the role of prenatal exposure to infection on offspring inflammatory profiles is unexplored. The aim was to study if maternal infections during pregnancy are associated with inflammation among offspring in later life and to determine if there are sensitive periods of exposure.

**Methods:**

The study was comprised of 1719 participants in the Copenhagen Aging and Midlife Biobank (CAMB) who were also members of the Copenhagen Perinatal Cohort (CPC). When the CPC was established, information on maternal infections during pregnancy was prospectively collected by a trained medical doctor. The inflammatory measures collected in late midlife included, C-reactive protein (CRP), Interleukin-6 (IL-6), TNF-alpha (TNF-α) and Interleukin-10 (IL-10). Multivariable ordinary least squared regression models were implemented to explore associations between maternal infection and inflammatory measures in offspring, controlling for maternal smoking, pre-pregnancy body mass index, age, marital status and parity.

**Results:**

Maternal infection was associated with a 7% lower CRP level (95% CI, − 17,5%) among offspring compared with offspring born to women without an infection and similarly an 8% lower level of IL-6 (95% CI -15,1%), and a 9% lower level of IL-10 (95% CI, − 23,20%). However, differences did not reach significance. The effects of infection during the first trimester did not differ from infections later in the pregnancy.

**Conclusions:**

Our results suggested that prenatal exposure to infection may be associated with lower levels of inflammatory markers among adult offspring. Additional prospective studies are needed to further explore this finding.

## Background

Recent studies indicate that chronic inflammation is implicated in a host of aging-related conditions [1–3], and may be a mechanism that links early life exposures with later health [4]. The prenatal and early environment may influence the regulation of inflammation and inflammatory phenotypes through the process of *developmental plasticity* [5]. Developmental plasticity provides organisms with the ability to change function and structure in response to environmental cues such as early nutritional and microbial exposures, typically during critical periods, such as the fetal period and infancy.

Maternal health may directly affect the fetal environment through factors such as subclinical maternal inflammation or even maternal infection or illness [6, 7]. Many common maternal bacterial or viral infections such as urinary tract and respiratory infections have been shown to be associated with preterm birth [6]. Goldenberg et al. and Romero et al. have shown that maternal inflammation exposed the fetus to an increased expression of cytokines, chemokines and lipid mediators through circulation, suggesting that common infections may play a role in the development of inflammation [8, 9]. To our knowledge, no previous studies have investigated the association between maternal infections and inflammation among adult offspring.

It is unclear how infections early in life affect later inflammation. The *hygiene hypothesis* posits that exposure to infectious agents protects against a large spectrum of disorders related to immune dysregulation, as evidenced by increasing rates of the full spectrum of atopic illnesses, which have risen in parallel to improved sanitary conditions [10, 11]. In support of the hygiene hypothesis, McDade et al. have shown that microbial exposures during the first two years of life were associated with lower levels of C-reactive protein (CRP) in adulthood [12]. This was the only observational study identified addressing the association between infant microbial exposures and later inflammation. The aim of the current paper was to study if maternal bacterial or viral infections during pregnancy are associated with inflammation among offspring in later life. Furthermore, we aimed to determine if exposure to infections during the first trimester of pregnancy is a particular sensitive period in relation to the inflammatory profile of offspring in late midlife.

## Materials and methods

The study sample was comprised of participants in the Copenhagen Aging and Midlife Biobank (CAMB) [13] who were also members of the Copenhagen Perinatal Cohort (CPC). [14**]** The CPC consists of 9125 consecutive deliveries by 8949 pregnant women giving birth at the Copenhagen University Hospital from October 1959 through December 1961, with 8400 infants surviving the first month after birth [14]. In 2009–2011, all CPC participants who were alive and living in eligible study areas (*n* = 5196) were invited to participate in CAMB. A total of 1719 agreed to participate in the clinical examination, including blood draw, constituting a 33% participation rate. The CAMB received ethical approval from the Scientific Ethics Committee Denmark (Protocol # H-A-2008-126).

A single physician was responsible for collecting maternal information through interviews with the mothers at the first antenatal visit and five days following delivery. A bacterial infection was considered present if there was a medical diagnosis and treatment was prescribed by a medical practitioner. The following bacterial infections were recorded: sinusitis, tonsillitis, pneumonia, cystitis, pyelonephritis, bacterial venereal infection or other bacterial infection. A viral infection was judged present if (1) a medical diagnosis was made by a general practitioner or (2) symptoms consistent with minor respiratory illness or influenza were present and accompanied by maternal bed rest and a temperature of ≥38 °C. The time during pregnancy in which the infection occurred was reported by trimester. We categorized infections as no infection during pregnancy versus any number of viral or bacterial infections at any time during pregnancy. To test our hypothesis that the first trimester of pregnancy is a particular sensitive period in relation to the inflammatory profile of offspring in late midlife we further categorized the infection data as no infection during pregnancy, having at least one infection during the first trimester of pregnancy but not during other periods, having at least one infection during the second or third trimester of pregnancy but not the first trimester, or having an infection in the first trimester and in the second/and or third trimester in the pregnancy This analysis is based on 1705 participants with available information on the timing of infections in pregnancy.

CRP, IL-6, IL-10 and Tumor necrosis factor α (TNF-α) were included in the current study. The inflammatory markers were derived from non-fasting blood samples collected at the CAMB in 2009 to 2011. Cytokines were measured in plasma with EDTA as the anticoagulant and analyzed individually as continuous variables. High sensitivity CRP was analyzed by Roche/Hitachi MODULAR P, with a measuring range of 0.1–20 mg/L (0.95–190 mmol/L at the Clinic Biochemical Laboratory and IL-6, TNF-α and IL-10 were analyzed in EDTA plasma by electro-chemiluminiscence multiplex system on a Sector 2400 Imager at the Centre of Inflammation and Metabolism, Rigshospitalet. The Lower limit of detection (LOD) for IL-6 was 0.21 pg/ml and inter assay coefficient of variation (CV) was 11 -21%. For IL-10 LOD =0.21 pg/ml and inter assay CV was 15–28%. For TNF-α LOD was 0.28 pg/ml and inter assay CV was 9 -13%. There were 0.4 and 6.5% of values below the LOD for IL-6 and IL-10 respectively, and these values were substituted using simple imputation.

Potential confounders include maternal age, parity, maternal tobacco smoking during pregnancy, maternal pre-pregnancy body mass index (BMI), derived from self-reports of height and weight collected during pregnancy, and maternal marital status categorized as married versus unmarried or other.

The inflammatory markers were analyzed as continuous variables and transformed using the natural logarithm to approach normality. Thus, regression coefficients can be interpreted as the relative increase (factor) in the outcome due to changes in the exposure. Crude linear regression coefficients show the association between increases in continuous covariate measures on the inflammatory markers. Geometric means of the inflammatory markers by categories of categorical covariates were performed. A series of ordinary least squares regression models with infections as the exposure and each inflammatory marker as the outcome were performed. The first model adjusted for maternal age at birth and parity, the second model additionally adjusted for maternal smoking, pre-pregnancy BMI and marital status. The same analyses stratified by timing of the infection were also carried out. Missing data was handled using a chained equation multiple imputation model with 10 imputations [15].

## Results

The offspring were between the age of 49 and 53 years at blood draw, 56% were female, the mean body mass index was 26 (SD 4.7) and 30.6% had an upper secondary education with only 2% of participants reporting no education. (results not shown) The mean age of the mothers at conception was 26.7 (SD 6.6) and 23% had an infection during pregnancy. Other baseline characteristics are shown in Table 1. Characteristics of the study population stratified by maternal infection during pregnancy are presented in Table 2. Maternal pre-pregnancy BMI was not associated with any of the inflammatory markers of interest and maternal age at birth was negatively associated with CRP but not with the other inflammatory markers in a crude model (Table 3). Increasing parity was positively associated with the inflammatory markers. Among women with an infection, the geometric means of the offspring inflammatory markers were lower than among women without an infection. There were higher levels of inflammatory markers among the offspring of women who were unwed at conception in comparison with married women, particularly in CRP and IL-6. There were not considerable differences in offspring inflammatory levels among smoking and non-smoking mothers or among males and females, with the exception of higher levels of IL-10 among male (geometric mean = 4.66) vs. female (geometric mean = 4.22) offspring.

**Table 1:** Characteristics of Danish men and women with continuous participation in the Copenhagen Perinatal Cohort and the Copenhagen Aging and Midlife Biobank

	N	Mean (SD and range)/percent
Maternal pre-pregnancy BMI (kg/m^2^)*	1616	21.7 (2.9; 14.7–47.8)
Maternal age at birth (years)	1709	26.7 (6.6; 14.6–48.1)
C-reactive protein	1692	2.35 (4.8; 0.01–108)
Interleukin-6	1689	2.7 (11.6; 0.01,442)
Interleukin-10	1681	8.9 (42; 0.01–509)
Tumor necrosis factor α	1691	5.3 (8.1; 0.65–208)
Parity		
1	822	48
2	470	27
3	263	15
4	112	7
5+	51	3
Infection during pregnancy		
No	1313	77
Yes	392	23
Maternal civil status at conception		
Married mother	1167	68
Unmarried mother	542	32
Tobacco smoking in pregnancy		
No	892	52
Yes	801	47
Child’s gender		
Male	753	44
Female	966	56

Means and standard deviations of continuous variables and frequency and percent of categorical variables

*BMI* body mass index

**Table 2:** Characteristics of participants by exposure to prenatal infection

	No infection during pregnancy *N* = 1313	Infection during pregnancy *N* = 392	*P*-Value*
Maternal pre-pregnancy BMI (kg/m2)	21.7	21.5	0.284
Maternal age at birth (mean years)	26.8	26.3	0.191
Parity n (%)			
1	622(47)	196 (50)	
2	370 (28)	97 (25)	
3	194 (15)	63 (16)	
4	83 (6)	29 (7)	
5+	43 (3)	7 (2)	0.299
Maternal civil status at conception n(%)			
Married mother	889 (68)	275 (70)	
Unmarried mother	422 (32)	117 (30)	0.382
Tobacco smoking in pregnancy n(%)			
No	674 (52)	203 (53)	
Yes	615 (48)	183 (47)	0.917
Child’s gender n(%)			
Male	742 (57)	217 (55)	
Female	571 (43)	175 (44)	0.686

Means or geometric means and standard deviations are presented for the continuous variables and number and percent for the categorical variables together with *p*-values

*calculated with ANOVA for continuous variables and chi^2^ for categorical variables

**Table 3:** Crude linear regression coefficients showing the association between increases in continuous covariates on inflammatory markers

	Continuous variables/Geometric mean			
	CRP	IL-6	IL-10	TNF-α
Maternal pre-pregnancy BMI (kg/m^2^)	1.02 (1.00,1.03)	1.01 (1.00,1.02)	1.00 (1.00,1.01)	1.00 (0.97,1.02)
Maternal age at birth (years)	0.99 (0.98,0.99)	1.00 (0.99,1.00)	1.00 (1.00,1.00)	1.00 (0.99,1.01)
Parity				
1	1.20	1.65	4.48	1.15
2	1.12	1.60	4.39	1.22
3	1.21	1.70	4.31	1.35
4	0.91	1.68	4.35	1.17
5+	1.63	2.14	5.16	1.02
*p*-value for difference	0.06	0.01	0.09	0.56
Infection during pregnancy				
No	1.20	1.68	4.44	1.22
Yes	1.09	1.54	4.39	1.12
*p*-value for difference	0.18	0.06	0.53	0.28
Maternal civil status at conception				
Married mother	1.03	1.60	4.39	1.19
Unmarried mother	1.35	1.78	4.53	1.19
*p*-value for difference	< 0.001	0.001	0.22	0.90
Tobacco smoking in pregnancy				
No	1.13	1.63	4.39	1.22
Yes	1.21	1.68	4.48	1.15
*p*-value for difference*	0.25	0.45	0.27	0.42
Child’s gender				
Male	1.15	1.67	4.66	1.22
Female	1.20	1.65	4.22	1.17
*p*-value for difference*	0.48	0.93	< 0.001	0.64

Geometric means by categories of categorical covariates with corresponding *p*-values

*BMI* body mass index*, CRP* C-reactive protein*, IL-6* Interleukin-6*, IL-10* Interleukin-10, *TNF-α* tumor necrosis factor-α, *p-values obtained using ANOVA

Maternal infection during pregnancy was associated with a 7% lower CRP level (among offspring compared with offspring born to women without an infection (95% CI, − 17,5%) and similarly an 8% lower level of IL-6 (95% CI, − 15, 1%), and a 9% lower level of IL-10 (95% CI, − 23,20%) (Table 4). The confidence intervals are, however, broad and we cannot statistically confirm these findings. We did not find evidence of different effects during the first trimester, compared with later in the pregnancy (Table 3). For example, an infection only during the first trimester was associated with a 8% lower level of IL-6 (95% CI -25, 14%), whereas an infection only during the 2nd or third trimester was associated with a 7% lower level of IL-6 (95% CI, − 15,3%) and an infection in the first trimester and later in pregnancy was associated with 29% lower level of IL-6 (95% CI -49,0%) Adjustments for other prenatal factors did not significantly affect the estimates.

**Table 4:** Associations between maternal infection and timing of maternal infection during pregnancy and inflammatory markers

		CRP		IL-6		IL-10		TNF-α	
	Cases	Crude Factor (95% CI)	Factor (95% CI)*	Crude Factor (95% CI)	Factor (95% CI)*	Crude Factor (95% CI)	Factor (95% CI)*	Crude Factor (95% CI)	Factor (95% CI)*
Infection during pregnancy	392	0.93 (0.81,1.06)	0.93 (0.81,1.06)	0.92 (0.84,1.01)	0.92 (0.85,1.01)	0.91;(0.77,1.08)	0.91 (0.77,1.08)	0.99 (0.94,1.03)	0.99 (0.94,1.04)
Timing of infection									
Never	1313	1	1	1	1	1	1	1	1
Trimester 1	55	1.04 (0.78,1.34)	1.05 (0.80,1.38)	0.88 (0.73,1.06)	0.90 (0.74,1.07)	0.95 (0.85,1.06)	0.95 (0.66,1.36)	0.95 (0.85,1.06)	0.95 (0.85,1.06)
Trimester 2 &/or 3 Trimester 1 & later in pregnancy	317 20	0.90 (0.78,1.02) 0.90 (0.78,1.02)	0.90 (0.79,1.03) 0.90 (0.78,1.02)	0.92 (0.84,1.02) 0.90 (0.78,1.02)	0.93 (0.84,1.02) 0.90 (0.78,1.02)	0.99 (0.94,1.04) 0.90 (0.78,1.02)	0.91 (0.76,1.09) 0.90 (0.78,1.02)	0.99 (0.94,1.04) 0.90 (0.78,1.02)	0.99 (0.94,1.04) 0.90 (0.78,1.02)

The factors and 95% confidence intervals (CI) are presented. Analyses based on 1719 men and women with continuous participation in the Copenhagen Perinatal Cohort and the Copenhagen Aging and Midlife Bioank

*Controlled for parity, maternal age, maternal pre-pregnancy BMI and smoking in the last trimester, maternal civil status, gender

*BMI* body mass index*, CRP* C-reactive protein*, IL-6* Interleukin-6*, IL-10* Interleukin-10*, TNF-α* tumor necrosis factor-α

## Discussion

We found that increasing parity and being conceived by an unmarried mother, which was potentially a maternal stressor during pregnancy, were associated with higher levels of inflammatory proteins in middle age. In accordance with the *hygiene hypothesis*, our findings suggest that, with the exception of TNF-α, maternal viral and bacterial infections during pregnancy may be associated with lower levels of inflammation among adult offspring. We did not find evidence of a sensitive period of exposure to infections during the first trimester of pregnancy. The results, however, must be interpreted with caution due to the wide confidence intervals and insignificant estimates. In a previous study based on a Filipino population, McDade and colleagues found that higher levels of microbial exposure in infancy were associated with lower CRP levels in early adulthood [12]. They speculated that this finding might be due to early microbial exposures contributing to the development of anti-inflammatory regulatory networks representing plasticity in the development of anti-pathogen defenses [12]. The findings of the current study suggest that exposure to infection during pregnancy, in addition to early microbial exposures, may contribute to this plasticity.

To our knowledge, this is the first study of the association between prenatal infections and offspring inflammatory profiles in later life. Strengths of the study include the prospective design, detailed information on the timing of exposure and the ability to adjust for a number of potentially important prenatal confounders. There are, however, limitations; we do not have information about the severity of the infection in pregnancy. It would have been valuable to know if there were systemic signs of infection among the mothers, such as fever or leukocytosis or if the infection was localized, as one could postulate that systemic illness may have a greater effect than localized infection. The inflammatory markers were only measured at a single occasion. Furthermore, only 33% of those invited to CAMB participated in the clinical examination where physical testing and blood samples were collected. A comparison of CAMB participants with non-participants in Danish national registries suggests that non-participants were significantly more likely to die in the years following data collection than participants [15] suggesting that the CAMB population was in better health and this may have led us to underestimate the effect of prenatal infections on later inflammation, particularly if non-participants were also likely to have more prenatal infections than participants.

## Conclusion

We cannot make any definitive conclusions about the findings in this study, but it appears that prenatal exposure to infection may be associated with lower levels of inflammatory markers among adult offspring. This must be explored further in additional prospective studies, with more detailed information about the severity and type of infection.

## Abbreviations

CAMB: Copenhagen aging and midlife iobank
CPC: Copenhagen perinatal cohort
CRP: C-reactive protein
IL-10: Interleukin-10
IL-6: Interleukin-6
TNF-α: TNF-alpha
